# Segmental Arterial Mediolysis Associated with Renal Allograft Artery Dissection and Thrombosis During Kidney Transplantation

**DOI:** 10.3390/clinpract16060099

**Published:** 2026-05-24

**Authors:** Matteo Zanchetta, Natale Calomino, Giuseppe Ietto, Vanessa Borgogni, Giorgio Micheletti, Sergio Antonio Tripodi, Daniele Marrelli, Franco Roviello, Gian Luigi Adani

**Affiliations:** 1 Unit of General Surgery and Surgical Oncology, Department of Medicine, Surgery and Neurosciences, Siena University Hospital, Viale Mario Bracci 16, 53100 Siena, Italy; 2 Kidney Transplant Unit, Siena University Hospital, Viale Mario Bracci 16, 53100 Siena, Italy; 3 General, Emergency and Transplant Surgery Department, Ospedale di Circolo e Fondazione Macchi, ASST Settelaghi, 21100 Varese, Italy; 4 Pathology Unit, Department of Oncology, Siena University Hospital, Viale Mario Bracci 16, 53100 Siena, Italy

**Keywords:** segmental arterial mediolysis, kidney transplant, graft, vascular surgery, renal artery, arteriopathy, transplantation, thrombosis, dissection, emergency surgery

## Abstract

**Background**: Segmental arterial mediolysis (SAM) is a rare, non-inflammatory, non-atherosclerotic, non-hereditary arteriopathy of unknown etiology that typically affects medium-sized visceral arteries. The absence of reliable diagnostic criteria poses a significant challenge. Consequently, the diagnosis of SAM should be considered in the setting of a distinctive combination of clinical features, angiographic findings, and histopathology. Renal artery involvement is uncommon, and its occurrence in the donor graft during kidney transplantation (KT) has not previously been reported. **Case presentation**: We report the case of a kidney graft from a deceased donor in her seventh decade of life, transplanted into a recipient in her seventh decade of life. Donor–recipient ABO compatibility was confirmed, and both complement-dependent cytotoxicity crossmatch and flow cytometry crossmatch were negative. Cold ischemia time was 14 h, and warm ischemia time was 20 min. Immediately after declamping, massive thrombosis of the graft renal artery was observed and confirmed using an intraoperative flowmeter. The arterial anastomosis was taken down, the thrombus was removed, the artery was flushed with heparin, and the anastomosis was reconstructed using interrupted sutures. Despite revision, no arterial flow was detected, and the graft was deemed unsalvageable and explanted. Histopathological examination showed thinning of the tunica media, reduced smooth muscle cells on desmin staining, medial-adventitial dissection, and occlusive thrombosis, findings considered likely attributable to SAM. **Conclusions**: This case suggests that occult donor arterial wall disease compatible with SAM may present catastrophically during KT and may lead to immediate graft loss despite standard surgical salvage attempts. Although no validated strategy currently exists to screen for or prevent occult SAM in asymptomatic donors, awareness of this entity may assist transplant surgeons and pathologists in the evaluation of unexplained early graft arterial thrombosis, donor-graft vascular pathology, and communication with centres receiving paired organs from the same donor.

## 1. Introduction

Segmental arterial mediolysis (SAM) is a rare, non-inflammatory, non-atherosclerotic and non-hereditary arteriopathy of unknown aetiology, typically affecting medium-sized abdominal visceral arteries, with occasional involvement of retroperitoneal, intracranial and coronary arteries [1,2]. Initially, SAM was described in different terms, but the name “segmental arterial mediolysis” was later adopted to reflect the absence of inflammatory features [3,4,5].

SAM was previously considered a possible precursor of fibromuscular dysplasia (FMD) because of shared histological features [5,6]. However, it is now generally regarded as a distinct clinical entity. Its pathological hallmark is mediolysis, characterized by vacuolization and lysis of smooth muscle cells in the arterial media [7]. This process may lead to separation of the media from the adventitia, weakening of the arterial wall, and subsequent aneurysm formation, dissection, stenosis, thrombosis, occlusion, or arterial rupture [8]. The most common clinical presentation is abdominal pain, usually related to dissections or aneurysms [1].

To date, no genetic predisposition has been identified, and the initiating event leading to arterial injury remains unclear. Definitive diagnostic criteria are also lacking. Because SAM may be clinically silent until macroscopic vascular damage occurs, early diagnosis before acute presentation may be unattainable. Management must therefore be individualized, ranging from conservative treatment in mild or self-limiting cases to endovascular or surgical intervention in severe or life-threatening presentations.

Renal artery involvement in SAM has been reported; however, to our knowledge, this is the first reported case involving the renal artery of a donor kidney graft during kidney transplantation (KT). In this case, occult donor-derived arterial wall disease became clinically evident only after graft reperfusion and intraoperative vascular manipulation, resulting in immediate renal allograft loss due to renal artery dissection and thrombosis. Histopathological examination of the explanted graft artery showed degenerative medial changes likely attributable to SAM. This case highlights the diagnostic challenges of this condition, its relevance for transplant surgeons and pathologists, and the importance of considering donor-derived vascular pathology in cases of unexplained early graft arterial thrombosis.

## 2. Case Report

The renal graft donor was a woman in her seventh decade of life whose brain death was declared following a massive cerebrovascular accident; the donation was therefore classified as donation after brain death. Her body mass index was 23.9 kg/m^2^. Her medical history was notable for long-standing arterial hypertension, which was pharmacologically managed. Donor serology was positive for IgG against cytomegalovirus (CMV). Pre-procurement abdominal ultrasound (US) did not show any significant urological or vascular abnormality. A preoperative donor CT scan was not performed, as it was not part of the routine pre-harvesting evaluation. The graft Karpinski score was 1, with a single point from the interstitial component of the scoring system. Donor–recipient ABO compatibility was confirmed, and both complement-dependent cytotoxicity crossmatch and flow cytometry crossmatch were negative.

The recipient was a woman in her seventh decade of life with end-stage chronic kidney disease secondary to autosomal dominant polycystic kidney disease. She was receiving haemodialysis three times weekly through a surgical arteriovenous fistula in the left upper arm and maintained residual diuresis of approximately one litre per day. Her medical history included bronchial asthma, chronic obstructive pulmonary disease, an episode of optic neuritis, neurofibromatosis without identified genetic mutations, arterial hypertension, hysterectomy for uterine fibroids, tonsillectomy, and appendectomy. Recipient serology was positive for anti-CMV IgG. Preoperative immunosuppression consisted of basiliximab and methylprednisolone.

A standard KT was performed in the right iliac fossa. The graft had a single renal artery and a single renal vein. Cold ischemia time was 14 h, and warm ischemia time was 20 min. No macroscopic calcification or obvious vascular abnormality of the graft renal artery was observed during back-table preparation or implantation. A standard atraumatic vascular clamp routinely used for KT was applied. Immediately after declamping, thrombosis of the graft renal artery was observed and confirmed by intraoperative flowmeter assessment. The arterial anastomosis was therefore taken down, the thrombus was removed, and the artery was flushed with heparinized solution. The arterial anastomosis was then reconstructed using interrupted sutures. Despite anastomotic revision, no arterial flow was detected. In view of the persistent absence of perfusion and massive renal artery thrombosis, the graft was deemed unsalvageable and was explanted.

The initial intraoperative differential diagnosis included a technical anastomotic complication, intimal dissection related to surgical manipulation, and, less likely, a recipient-related prothrombotic condition. However, the persistent absence of flow after thrombectomy, heparinized flushing, and reconstruction of the arterial anastomosis made an isolated technical anastomotic cause less likely.

Postoperatively, US examination excluded perirenal fluid collections and urinary tract dilatation. Nephrology, vascular surgery, and haematology consultants were involved in the postoperative assessment, and specific blood tests were performed. The recipient had no history of thromboembolism, and testing was negative for antiphospholipid syndrome or similar prothrombotic disorders.

**Figure 1:**
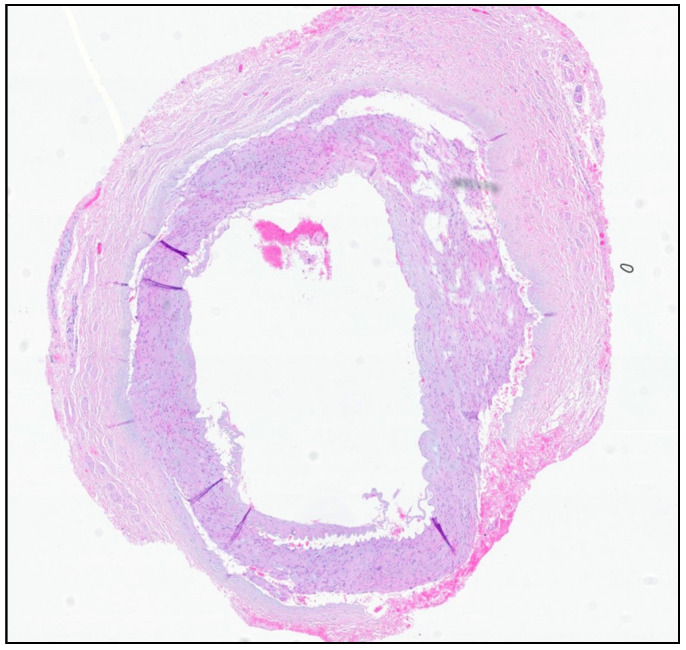
Whole arterial section (magnification 20×, hematoxylin and eosin staining): a severe non-inflammatory medial damage with gaps in the outer layer extending towards the adventitia is shown.

**Figure 2:**
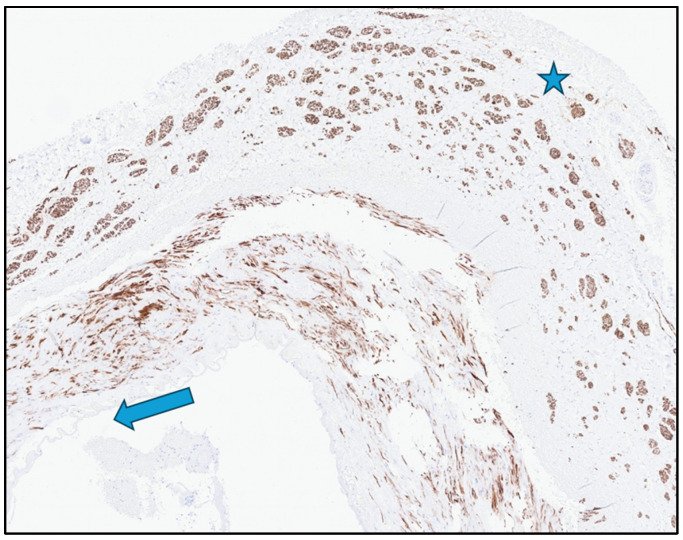
Arterial wall (40× magnification, immunohistochemistry anti-desmin). Desmin staining (brown) highlights the decrease of smooth muscle cells within the tunica media of renal artery. There is also a relative increase of smooth muscle bundles in the adventitia (star). The arrow indicates the internal elastic lamina.

Histopathological examination of the explanted graft artery showed thinning of the tunica media, reduced smooth muscle cells on desmin staining, dissection between the tunica media and adventitia, and occlusive thrombosis [Figure 1 and Figure 2]. These findings supported a diagnosis of degenerative medial pathology of the graft renal artery, likely attributable to SAM. Histopathological examination suggested that the degenerative arterial wall changes involved most of the graft renal artery and were not limited exclusively to the clamped segment. Therefore, intraoperative mechanical stress from vascular clamping or manipulation may have acted as a precipitating factor in an already structurally compromised arterial wall.

The recipient had an uneventful postoperative recovery and remained on the waiting list for a new compatible graft. Because the contralateral kidney from the same donor had been allocated for transplantation at another institution, that centre was notified as soon as the histopathological diagnosis compatible with SAM was made. This communication was intended to allow enhanced vigilance for possible donor-derived vascular abnormalities in the contralateral graft and to support stricter imaging follow-up beyond routine post-transplant monitoring.

## 3. Discussion

This case describes an exceptionally rare event of immediate intraoperative renal allograft loss following renal artery dissection and thrombosis in a donor graft artery with histopathological features likely attributable to SAM. The event occurred immediately after declamping, and perfusion could not be restored despite thrombectomy, heparinized flushing, and reconstruction of the arterial anastomosis. The case is relevant because occult donor-derived arterial wall disease may remain undetected during routine donor assessment and become clinically evident only after reperfusion or intraoperative vascular manipulation.

The diagnosis of SAM remains challenging because no universally accepted diagnostic criteria or specific serum or genetic biomarkers are available. In many cases, diagnosis relies on a combination of clinical presentation, imaging findings, exclusion of mimicking vasculopathies, and histopathology when tissue is available. In the present case, tissue from the explanted graft artery allowed direct pathological assessment. Histopathology showed thinning of the tunica media, reduced smooth muscle cells on desmin staining, medial-adventitial dissection, and occlusive thrombosis, supporting a diagnosis likely attributable to SAM. We acknowledge that intraoperative mechanical injury may have acted as a precipitating factor in an already structurally compromised artery.

A major diagnostic challenge is distinguishing SAM from other vascular disorders, particularly fibromuscular dysplasia, hereditary connective tissue diseases, vasculitides, and mycotic aneurysms [9,10,11]. This distinction is often difficult because tissue sampling is not usually feasible, as the affected arteries are frequently deep abdominal vessels [12]. SAM and fibromuscular dysplasia share some histological features, and SAM was previously considered by some authors to represent a variant or precursor of fibromuscular dysplasia [13,14]. However, fibromuscular dysplasia more commonly affects younger women, smokers, and the renal or carotid arteries, usually causing stenosis and only rarely rupture [15]. Conversely, SAM more often affects older adults and typically presents with aneurysms or dissections involving the celiac and superior mesenteric arterial territories. The absence of a serum or genetic biomarker remains a significant limitation. Kalva et al. proposed clinical, radiological, and serological diagnostic criteria based on the exclusion of congenital predisposition or inflammatory arteritis, the presence of arterial dissection, fusiform aneurysm, occlusion, beaded appearance, or wall thickening of mesenteric or renal arteries, and the absence of elevated inflammatory markers [16]. Skeik et al. further suggested that SAM should be diagnosed in the presence of multiple arterial pathologies or multiple lesions within one artery, to distinguish it from an isolated aneurysm or dissection [17]. In clinical practice, diagnosis is usually based on characteristic vascular abnormalities on CTA, MRI, or catheter angiography, together with exclusion of mimicking diseases and histopathological confirmation when available [12]. CTA is often considered the preferred imaging modality because it provides adequate spatial resolution for detecting aneurysms, dissection, stenosis, occlusion, and organ ischemia, and may also be useful for follow-up [16,18]. In our case, the availability of histopathological tissue from the explanted graft artery provided direct diagnostic support, while the absence of recipient thrombophilia and the failure to restore flow despite surgical revision made isolated technical or coagulative causes less likely.

The underlying cause of SAM remains unclear. Proposed mechanisms include repetitive vasoconstrictive injury, hypoxia related to comorbidities, and physical stress [4,19]. These hypotheses are supported by the observation that arteries exposed to chronic vasospasm may show histological features similar to those observed in SAM [18]. In our case, intraoperative clamping or vascular manipulation may have acted as a precipitating factor in a donor renal artery already structurally compromised by degenerative medial disease. Regardless of the initial trigger, SAM is generally described as progressing through an injurious phase and a reparative phase, with characteristic lesions including mediolysis, separation or tear, arterial gaps, and reparative granulation or fibrosis [1,7,20]. Mediolysis, the hallmark lesion, begins with vacuolization and lysis of smooth muscle cells in the outer media of the arterial wall [7,21]. This medial degeneration may lead to separation of the media from the adventitia, segmental disruption of the arterial wall, and subsequent aneurysm formation, dissection, stenosis, thrombosis, occlusion, or rupture [8,12,22]. In elderly patients, age-related degeneration of adventitial stromal connections may further facilitate medial-adventitial separation [23]. As the lesion progresses, destruction of the internal elastic lamina and intima may allow blood to dissect into the arterial wall, promoting vessel instability, intramural hematoma, dissecting aneurysm, luminal thrombosis, and arterial rupture [8,20]. These mechanisms are consistent with the findings observed in our graft artery, namely medial thinning, medial-adventitial dissection, and occlusive thrombosis.

The clinical presentation of SAM depends on the extent of arterial wall injury, the vascular territory involved, and the timing of disease progression. It may present acutely with catastrophic vascular events or follow a more insidious, sometimes self-limiting course. SAM has been described in all age groups, but it mainly affects adults and elderly individuals, with a slight male predominance [24]. Although it typically involves medium-sized splanchnic arteries, renal, spinal, intracranial, ovarian, and iliac arterial involvement has also been reported [25,26,27,28,29]. Abdominal SAM most commonly presents with abdominal pain, ranging from chronic discomfort to acute abdomen with hemoperitoneum and haemorrhagic shock [30,31]. However, asymptomatic cases with incidentally detected vascular abnormalities have also been described [32]. In a systematic review of 143 cases, the most frequently affected vessels were the superior mesenteric, hepatic, celiac, renal, and splenic arteries, with renal artery involvement reported in 25.9% of cases [17]. Multiple arterial involvement was present in more than 62% of patients [17]. The most common vascular findings were aneurysm, dissection, and rupture, followed by stenosis, occlusion, thrombosis, pseudoaneurysm, and wall thickening [17]. Arterial occlusion, often associated with underlying dissection, may result in end-organ ischemia, including renal infarction or bowel ischemia [12]. In this context, our case represents an unusual transplant-related manifestation of SAM, presenting as immediate graft renal artery dissection, thrombosis, and loss of perfusion.

In retrospect, preoperative CT or CT angiography might have provided additional information regarding macroscopic vascular abnormalities of the graft renal artery, such as calcifications, aneurysmal changes, stenosis, or dissection. However, the donor had no known diagnosis of systemic vasculopathy, and preoperative abdominal US did not show significant vascular abnormalities. Furthermore, occult SAM may remain undetectable before an acute vascular manifestation, particularly in the absence of gross arterial changes. Therefore, although CT/CTA may be considered when donor vascular disease is suspected or when abnormal vascular findings are encountered during procurement or back-table preparation, routine SAM-specific screening of asymptomatic donors cannot currently be recommended.

From a practical transplant perspective, this case does not support routine SAM-specific screening in asymptomatic donors, as no validated clinical, serological, genetic, or imaging criteria are currently available to identify occult SAM before an acute vascular manifestation. Nevertheless, awareness of this condition may be relevant during organ procurement, back-table preparation, and implantation, particularly when organs are obtained from elderly donors or donors with vascular risk factors. Vascular complications during kidney transplantation may be influenced by graft arterial anatomy and technical factors, and meticulous vascular handling remains essential, particularly when dealing with complex or potentially fragile donor vessels. Previous experience with complex renal graft arterial anatomy has emphasized the importance of careful vascular reconstruction and preservation of graft perfusion during kidney transplantation [33]. Although mechanical stress cannot be proven as the sole cause of the vascular event in the present case, gentle handling of donor vessels, avoidance of excessive traction or torsion, careful selection of the clamping site, minimization of repeated clamping, and use of atraumatic vascular clamps may represent reasonable precautions when dealing with potentially fragile arterial walls. These measures should not be interpreted as SAM-specific prevention, but as general surgical principles aimed at reducing the risk of arterial injury in structurally compromised vessels. In the event of unexplained donor-graft arterial dissection or thrombosis, prompt histopathological assessment and communication with centres receiving paired organs from the same donor are advisable.

Because the lesion in our case involved the donor renal artery, we do not believe that SAM-specific precautions are required for the recipient during future transplantation. The event should, however, be clearly documented, and standard pre-transplant vascular and thrombophilia assessment should be performed according to institutional protocols. In the absence of recipient vasculopathy or prothrombotic disorders, this event alone should not be regarded as a contraindication to a new transplantation.

No standardized guidelines for the treatment of SAM currently exist, and management should be individualized according to clinical presentation, lesion location, severity, and comorbidities. Strict control of arterial hypertension is generally considered important to reduce further arterial wall stress [34,35]. Accurate distinction from inflammatory vasculopathies is also essential because corticosteroids and immunosuppressive agents used for vasculitis do not benefit SAM and may expose patients to unnecessary risks, including infection, impaired wound healing, and worse outcomes [36,37]. The use of antiplatelet or anticoagulant therapy remains controversial and should be individualized, given the potential coexistence of thrombotic complications and risk of aneurysmal rupture. Many mild presentations may follow a benign or self-limiting course, and spontaneous regression after conservative management has been reported [18,38]. In the retrospective analysis by Naidu et al., most patients showed stability or regression during follow-up after surviving the acute phase [24]. Conversely, patients presenting with haemodynamic instability, intra-abdominal haemorrhage, progressive lesions, or end-organ ischemia may require urgent endovascular or surgical intervention [30,39]. The management of asymptomatic aneurysms remains controversial, although treatment may be considered for lesions at higher risk of rupture, particularly colic artery aneurysms, for which endovascular treatment is often considered first [40,41,42].

The true prevalence of SAM is probably underestimated, partly because some patients may have subclinical disease, awareness of this rare entity remains limited, and angiography or CTA is not routinely performed in all patients with abdominal pain [8,42]. Although recent retrospective analyses have reported mortality rates of 4.3–7%, lower than those described in older series, SAM may still present with catastrophic vascular events [1,17]. For patients who survive the acute phase, subsequent stability or even disease regression has been reported [18]. Nevertheless, the optimal follow-up strategy remains unclear because of the rarity of the disease and the lack of large-scale prospective data. Several authors have suggested close imaging surveillance during the first year, in some cases every three months, followed by at least annual imaging monitoring with CTA, angiography, or MRI, together with strict blood pressure control [9,10,16,43,44].

In our case, follow-up considerations were particularly relevant because the contralateral kidney from the same donor had been transplanted at another institution. Given the possibility of multifocal or bilateral renal artery involvement in SAM [35], the other centre was promptly notified after the histopathological diagnosis, so that enhanced clinical and imaging surveillance of the contralateral graft could be considered. This reinforces the importance of inter-centre communication when donor-derived vascular pathology is identified.

## 4. Conclusions

Segmental arterial mediolysis is a rare, potentially underreported arteriopathy that may remain clinically silent until acute vascular injury occurs. The absence of reliable diagnostic criteria poses a significant challenge, sometimes resulting in delays with severe consequences. We report a rare case of intraoperative graft loss during kidney transplantation following renal artery dissection and thrombosis, with histopathological features of the graft renal artery likely attributable to SAM. This case highlights that occult donor-derived arterial wall disease may become evident only after graft reperfusion or vascular manipulation and may lead to graft loss despite standard surgical salvage attempts. Although no validated SAM-specific screening or preventive strategy is currently available for asymptomatic donors, transplant surgeons should consider donor-derived arterial pathology in cases of unexplained immediate graft arterial thrombosis. Histopathological assessment, prompt communication with centres receiving organs from the same donor, and rigorous follow-up of the contralateral transplanted graft may be clinically important. Finally, this case underscores the importance of raising awareness of this rare pathology among transplant surgeons and pathologists, as early recognition may influence diagnostic assessment, inter-centre communication, and post-transplant surveillance.

## Data Availability

The original contributions presented in this study are included in the article. Further inquiries can be directed to the corresponding author.
